# Origin and Evolution of the Eukaryotic SSU Processome Revealed by a Comprehensive Genomic Analysis and Implications for the Origin of the Nucleolus

**DOI:** 10.1093/gbe/evt173

**Published:** 2013-11-07

**Authors:** Jin-Mei Feng, Hai-Feng Tian, Jian-Fan Wen

**Affiliations:** ^1^State Key Laboratory of Genetic Resources and Evolution, Kunming Institute of Zoology, Chinese Academy of Sciences, Kunming, Yunnan, China

**Keywords:** SSU processome, evolution, nucleolus, LECA, origin

## Abstract

As a nucleolar complex for small-subunit (SSU) ribosomal RNA processing, SSU processome has been extensively studied mainly in *Saccharomyces cerevisiae* but not in diverse organisms, leaving open the question of whether it is a ubiquitous mechanism across eukaryotes and how it evolved in the course of the evolution of eukaryotes. Genome-wide survey and identification of SSU processome components showed that the majority of all 77 yeast SSU processome proteins possess homologs in almost all of the main eukaryotic lineages, and 14 of them have homologs in archaea but few in bacteria, suggesting that the complex is ubiquitous in eukaryotes, and its evolutionary history began with abundant protein homologs being present in archaea and then a fairly complete form of the complex emerged in the last eukaryotic common ancestor (LECA). Phylogenetic analysis indicated that ancient gene duplication and functional divergence of the protein components of the complex occurred frequently during the evolutionary origin of the LECA from prokaryotes. We found that such duplications not only increased the complex’s components but also produced some new functional proteins involved in other nucleolar functions, such as ribosome biogenesis and even some nonnucleolar (but nuclear) proteins participating in pre-mRNA splicing, implying the evolutionary emergence of the subnuclear compartment—the nucleolus—has occurred in the LECA. Therefore, the LECA harbored not only complicated SSU processomes but also a nucleolus. Our analysis also revealed that gene duplication, innovation, and loss, caused further divergence of the complex during the divergence of eukaryotes.

## Introduction

In all organisms, ribosome translates mRNAs into proteins and in doing so governs cell growth and survival. As pivotal components of ribosome, rRNA are transcribed and processed in the nucleolus of eukaryotes while in the cytoplasm of prokaryotes. Small-subunit (SSU) ribosomal RNA (SSU rRNA) is the sole RNA component of the small ribosomal subunit. In eukaryotes, the SSU rRNA (18S rRNA) gene is transcribed together with 5.8S and 28S (25S in yeast) rRNA genes into a common primary precursor rRNA (pre-rRNA), in which SSU rRNA is flanked by 5′ external transcribed spacer (5′ ETS) and internal transcribed spacer 1 (ITS1), then SSU rRNA is produced through U3 small nucleolar RNA (snoRNA)-mediated cleavages at A0 and A1 sites within 5′ ETS and A2 within ITS1 (Henras et al. 2008).

A large nucleolar ribonucleoprotein (RNP) complex called SSU processome was first identified to mediate this posttranscriptional processing in the yeast *Saccharomyces cerevisiae* (Dragon et al. 2002). To date, besides a known RNA component U3 snoRNA, which is present, though sometimes divergent, in all eukaryotes examined, the yeast SSU processome is known to consist of five small ribosomal subunit proteins and 72 nonribosomal proteins. All these yeast SSU processome proteins have been divided into 51 confirmed proteins, which include five ribosomal proteins and 46 nonribosomal proteins, and 26 probable proteins (Bernstein et al. 2004; Phipps et al. 2011). Previous studies revealed that at least 26 of the 51 confirmed proteins compose six subcomplexes (U3 snoRNP, Mpp10, U three protein A (UtpA), U three protein B (UtpB), U three protein C (UtpC), and Bms1/Rcl1), while the other 25 proteins do not belong to any subcomplexes (Phipps et al. 2011). 

Homologs to 72 of the *S. cerevisiae* SSU processome components have also been identified and characterized in the human genome and reported to form five similar subcomplexes (Granneman et al. 2003; Prieto and McStay 2007; Turner et al. 2009; Phipps et al. 2011). In crucifer plants, a snoRNP complex named NF D was thought to be competent for the primary cleavage in the 5′ ETS (Saez-Vasquez et al. 2004a, 2004b). In protist *Tryoanosoma cruzi*, homologs to *S. cerevisiae* SSU processome components were identified in silico (Nardelli et al. 2007). However, obviously, these previous studies are restricted to only a few organisms of a narrow range of taxa. Whether SSU processome is a ubiquitous SSU pre-rRNA processing mechanism in eukaryotes is accordingly still elusive.

Prokaryotes do not possess nuclei and nucleoli, and their SSU pre-rRNA processing mechanism is considered to be quite different from that in eukaryotes. Their pre-rRNA transcripts generally contain inverted repeats surrounding the SSU rRNA (16S rRNA) sequences and thus can form extended helical structures and contain the sites for the initial endonucleolytic cleavage and excision of pre-16S rRNA from the primary transcript, then the pre-16S rRNA is further processed to mature the 5′ and 3′ ends of 16S rRNA. In bacteria, a double-helix specific ribonuclease, RNase III, is responsible for releasing pre-16S rRNA from the primary transcript (Young and Steitz 1978; Britton et al. 2007), but how the 5′ and 3′ ends mature is still unclear. Till now, no homologs to *S. cerevisiae* SSU processome components have ever been reported to be involved in the biogenesis of 16S rRNA in bacteria. In archaea, a bulge-helix-bulge (BHB) endonuclease, which is not homologous to bacterial RNase III, is thought to be widely used to recognize and excise the BHB motif, which is inverted repeats surrounding the SSU (16S) rRNA and consists of two three-base bulges on opposite strands of the helix separated by four base pairs, to release pre-16S rRNA from the primary transcript, but how the 5′ and 3′ ends mature then is also unknown (Chant and Dennis 1986; Dennis et al. 1998; Tang et al. 2002). However, it was once reported that in a kind of Crenarchaeota, *Sulfolobus acidocaldarius*, a U3-like snoRNP that contains a U3-like snoRNA and five or six proteins, was shown to be responsible for the maturation of the 5′ end of the 16S rRNA (Potter et al. 1995; Russell et al. 1999). Later, homologs of seven of the *S. cerevisiae* SSU processome proteins were also found in several archaea (Mayer et al. 2001; Eschrich et al. 2002; Kuhn et al. 2002), and some homologs of snoRNAs were also found in archaea (Omer et al. 2000), though the previously reported U3-like snoRNA (Potter et al.1995) mentioned above was known to be an artifact later (Russell et al. 1997). These imply that the SSU pre-rRNA processing in archaea might be similar to that in *S. cerevisiae* at least in some aspects. Although some previous work showed that eukaryotic ancestral paralogous proteins, including some WD-40 domain-containing rRNA processosome proteins, were inherited from the last universal common ancestor (LUCA; Makarova et al. 2005), and eukaryotic proteins involved in the information-processing systems are of archaeal origin (Yutin et al. 2008), the origin of eukaryotic SSU processome, in its entirety, was never particularly and comprehensively explored. Consequently, a comprehensive genomic analysis of eukaryotic SSU processome component homologs in prokaryotes, especially in archaea, likely contains important insights into the evolutionary origin of the eukaryotic SSU processome.

Available genome databases of diverse organisms in the three domains of life are accumulating rapidly now, which provides excellent opportunities to address the aforementioned questions surrounding the SSU processome. In the present study, we used 77 completely sequenced genomes of various eukaryotes that were chosen as representatives of the five eukaryotic supergroups—opisthokonts, amoebozoa, plantae, excavates, and chromalveolates—to investigate the phylogenetic distribution of SSU processome in eukaryotes alongside prokaryotes, to explore the origin and evolution of the SSU processome, and surprisingly revealed some potential implications for the evolution of the nucleolus and even the eukaryotic cell.

## Materials and Methods

### Data Sources

Sequences of the 51 confirmed and 26 probable proteins of *S. cerevisiae* SSU processome were retrieved from the Saccharomyces Genome Database (SGD) (http://www.yeastgenome.org/, last accessed July 15, 2010) using their respective protein names. By using them as queries, the sequence data of putative proteins and genes of these components from 77 complete sequenced eukaryotes, representatives of the five eukaryotic supergroups listed in supplementary data S1 (Supplementary Material online), were downloaded from National Center for Biotechnology Information (NCBI) GenBank (http://www.ncbi.nlm.nih.gov/, last accessed November 26, 2013), EuPathDB (http://eupathdb.org/eupathdb/, last accessed November 26, 2013), Doe Joint Genome Institute (http://www.jgi.doe.gov/, last accessed November 26, 2013), Broad institute (http://www.broadinstitute.org/, last accessed November 26, 2013), and *Cyanidioschyzon merolae* Genome Project (http://merolae.biol.s.u-tokyo.ac.jp/, last accessed August 11, 2010). All the annotated proteins in 1,375 bacterial and 67 archaeal genome databases were downloaded from NCBI RefSeq database (Release 41, http://www.ncbi.nlm.nih.gov/refseq/, last accessed February 17, 2011).

### Homolog Search for the SSU Processome Proteins in the Three Domains of Life

With the 77 yeast SSU processome proteins mentioned earlier as queries, candidate eukaryotic homologs were obtained by using BlastP, Position-Specific Iterated Blast (PSI-Blast), and TBlastN search against the protein and gene databases from the 77 eukaryotic genomes with e-value less than 0.001. They were then assessed by using domain information in Pfam database 24.0 (http://pfam.sanger.ac.uk/, last accessed February 29, 2011) and reciprocal Blast search against the GenBank nonredundant (nr) protein database. Sequences lacking characteristic domains of the given proteins or shown to be other proteins were discarded. For any given eukaryotic genomes from which no homologs were detected to a SSU processome protein, the corresponding homologs obtained from the genomes of its closely related species were used as queries to search against its genome database to find homologs.

We searched against all 1,375 bacterial and 67 archaeal genome databases using the BlastP algorithm. All hits with an e-value less than 0.001 were collected. In addition, for some given proteins that had few archaeal homologs, all archaeal genomes were searched by using PSI-Blast for at least five iterations using the obtained top hits from archaea as queries, and the hits with e-value less than 1e−5 were retrieved. All obtained hits were further assessed using Pfam domain analysis and reciprocal Blast search as described earlier. Only those that passed these two analyses were accepted as putative homologs.

### Analysis of Functional Domain Composition of Eukaryote-Specific SSU Processome Proteins

SSU processome proteins with no homologs in prokaryotes were designated as eukaryote-specific SSU processome proteins, and their functional domain compositions were analyzed. The functional domain composition of a given protein was defined according to its domain repertoire. Protein domains were detected by searching against the Pfam database. All protein domains were divided into four groups according to the definition proposed by Staub et al. (2004): 1) ancient domain, to present in all the three domains of life; 2) archaeal domain, found in archaea and eukaryotes but not in bacteria; 3) bacterial domain, to be present in bacteria and eukaryotes but absent from archaea; 4) eukaryotic domains, to be specific to eukaryotes. Collectively, ancient, archaeal, and bacterial protein domains were called prokaryote-original protein domains.

### Multiple Sequence Alignment and Phylogenetic Analysis

According to the Blast search results for the homologs to yeast SSU processome proteins, single-gene phylogenies were reconstructed for each of those proteins that may have paralogs in eukaryotes to reveal the phylogenetic relationship among the paralogs. Additionally, to reveal the phylogenetic correlations between eukaryotic proteins and prokaryotic homologs, separate phylogenetic analyses were also performed for each of those proteins that have prokaryotic homologs. Finally, the homologous sequences from all 77 investigated eukaryotes, 14 archaea, and 10 bacteria, which are representatives selected from each phylum of prokaryotes used by Cox et al. (2008) were used to conduct the following phylogenetic analyses.

Multiple sequence alignment was performed using MUSCLE 3.8.31 (Edgar 2004) with default parameters. Nonhomologous insertions and sequence characters that could not be aligned with confidence were manually removed. Only unambiguously aligned regions were used for phylogenetic analysis. The best-fit models for each data set were selected by using ModelGenerator 0.85 (Keane et al. 2006). Maximum likelihood trees were constructed with both FastTree 2.1 (Price et al. 2010) using default CAT model of Stamatakis and other settings, and PhyML 3.0 (Guindon and Gascuel 2003) with 100 bootstrap replications. A gamma distribution split into four categories was used for consideration of the rate heterogeneity among sites.

Prior to the earlier phylogenetic analyses, we usually conducted preliminary analysis of the large data sets that included a great deal more bacterial similar sequences by using FastTree 2.1 with default parameters, and then only the sub-data sets including eukaryotic sequence data and the representative prokaryotes on the preliminary trees were picked out and subjected to the full analysis mentioned earlier.

## Results

### SSU Processome Proteins in Diverse Eukaryotes

The distribution of SSU processome proteins in the five eukaryote supergroups is summarized in table 1, and the phylogenetic distribution of these proteins in the diverse eukaryotic species investigated is shown in supplementary data S2 (Supplementary Material online). For the 46 nonribosomal proteins of the 51 confirmed yeast SSU processome proteins, we found that most of them have homologs in all the investigated species of the five eukaryotic supergroups, and only three proteins (Utp8, Utp9, and Utp16) are absent in almost all the lineages, except a subphylum of Fungi, Saccharomycotina, suggesting they are Saccharomycotina-specific, and several proteins (e.g., Utp17, Rrp7, Rrp36) are absent specifically in some lineages such as Kinetoplastid and Microsporidia, and some other proteins (e.g., Utp5, Utp10, Utp22, Rcl1, Utp2, Utp3, Utp20, Utp25, Rrp5, Sof1) are only absent specifically in a few or a single species. On the other hand, 24 of the 46 nonribosomal proteins were found to have two or more copies of homologs in 18 species. Interestingly, one of them, Nop1, was found to possess multiple homologs in all the investigated kinetoplastids. What is more important is that neither a given species nor a given lineage lacks all the nonribosomal proteins. There is no case where all the components of any of the six subcomplexes are absent in a given species or lineage; in fact, all the components of the four subcomplexes (U3 snoRNP, Mpp10, UtpB, and Bms1/Rcl1) are present in all eukaryotes, though three of their components are absent in a few species (e.g., Rrp9 is absent from *Giardia lamblia*, *Plasmodium knowlesi*, and *P. vivax*) or a single species (e.g., Utp18 is absent from *Thalassiosira pseudonana* and Rcl1 from *Trichomonas vaginalis*). Homologs to the five ribosomal proteins of the confirmed SSU processome proteins were observed in all the eukaryotes, and many species in each of the five major eukaryotic supergroups were found to have two or more copies of homologs to them.

**Table 1:** Summary of Distribution of the SSU Processome Proteins in the Five Eukaryotic Supergroups

Subcomplex	Protein Name	Opisthokonts	Amoebozoa	Plantae	Excavates	Chromalveolates	LECA
U3 snoRNP	Nop1	+	+	+	+	+	+
	Nop56	+	+	+	+	+	+
	Nop58	+	+	+	+	+	+
	Snu13	+	+	+	+	+	+
	Rrp9	+	+	+	+	+	+

Mpp10	Imp3	+	+	+	+	+	+
	Imp4	+	+	+	+	+	+
	Mpp10	+	+	+	+	+	+

UtpA	Utp4	+	+	+	+	+	+
	Utp5	+	+	+	+	+	+
	Utp8	+	−	−	−	−	−
	Utp9	+	−	−	−	−	−
	Utp10	+	+	+	+	+	+
	Utp15	+	+	+	+	+	+
	Utp17	+	+	+	+	+	+

UtpB	Utp1	+	+	+	+	+	+
	Utp6	+	+	+	+	+	+
	Utp12	+	+	+	+	+	+
	Utp13	+	+	+	+	+	+
	Utp18	+	+	+	+	+	+
	Utp21	+	+	+	+	+	+

UtpC	Rrp7	+	+	+	−	+	+
	Utp22	+	+	+	+	+	+
	Rrp36	+	+	+	+	+	+

*Bms1/Rcl1*	Bms1	+	+	+	+	+	+
	Rcl1	+	+	+	+	+	+

Unclassified	Utp2	+	+	+	+	+	+
	Utp3	+	+	+	+	+	+
	Utp7	+	+	+	+	+	+
	Utp11	+	+	+	+	+	+
	Utp14	+	+	+	+	+	+
	Utp16	+	−	−	−	−	−
	Noc4	+	+	+	+	+	+
	Utp20	+	+	+	+	+	+
	Utp23	+	+	+	+	+	+
	Utp24	+	+	+	+	+	+
	Utp25	+	+	+	+	+	+
	Dbp8	+	+	+	+	+	+
	Dhr1	+	+	+	+	+	+
	Dhr2	+	+	+	+	+	+
	Emg1	+	+	+	+	+	+
	Krr1	+	+	+	+	+	+
	Rok1	+	+	+	+	+	+
	Rrp3	+	+	+	+	+	+
	Rrp5	+	+	+	+	+	+
	Sof1	+	+	+	+	+	+

Confirmed ribosomal proteins	RPS4	+	+	+	+	+	+
	RPS6	+	+	+	+	+	+
	RPS7	+	+	+	+	+	+
	RPS9	+	+	+	+	+	+
	RPS14	+	+	+	+	+	+

Note.—“+” means the protein present in all or the majority of the member groups of a certain supergroup.

To sum up, the above results indicate that except for several *Saccharomycotina*-specific proteins and lineage-specifically absent proteins, most of the 51 confirmed SSU processome proteins were widely distributed in all extant eukaryotes. On the other hand, there is no case that either all nonribosomal proteins or all ribosomal proteins of the SSU processome are absent in a given lineage or species, and there is also no lineage or species in which all the components of a given subcomplex of the SSU processome are absent.

Our phylogenetic investigation also revealed that most of the 26 probable proteins are also widely distributed in the investigated eukaryotes (supplementary data S3 and S4, Supplementary Material online).

### Phylogenetic Distribution of SSU Processome Protein Homologs in Prokaryotes

Only four eukaryotic SSU processome proteins, Snu13, Imp3, Rps9, and Rps14, were found to have bacterial homologs: Imp3 and Rps9 both possess the common bacterial homologous protein Rps4; Snu13 and Rps14 have their respective bacterial homologs L7Ae and Rps11 (supplementary data S5, Supplementary Material online). However, the three bacterial homologous proteins are all common ribosomal proteins previously reported to exist in prokaryotes including archaea. No homologs to the nonribosomal proteins of eukaryotic SSU processome were found in bacteria at all.

In archaea, as many as 14 eukaryotic SSU processome proteins, including ten nonribosomal proteins (Nop1, Nop56, Nop58, Snu13, Imp3, Imp4, Utp23, Utp24, Emg1, Krr1) and four ribosomal proteins (Rps4, Rps6, Rps9, and Rps14), were found to have their homologs. Seven of them, Nop1, Nop56, Nop58, Snu13, Imp3, Imp4, and Emg1, were reported previously to have homologs in several archaea (Mayer et al. 2001; Eschrich et al. 2002; Kuhn et al. 2002), but these homologs were found in more archaeal species in this study. Altogether, we found that the 14 eukaryotic SSU processome proteins have 11 archaeal homologs—eight of them (Nop1, Snu13, Imp4, Emg1, Krr1, Rps4, Rps6, and Rps14) each have one corresponding archaeal homolog (Fibrillarin, L7Ae, Imp4, Nep1, Krr1, Rps4e, Rps6e, and Rps11), while Nop56 and Nop58 share a common archaeal homolog Nop56/58, Utp23 and Utp24 share archaeal Utp23/24, and Imp3 and Rps9 share archaeal Rps4 (table 2; for the distribution details in the investigated archaea see supplementary data S6, Supplementary Material online). The 14 eukaryotic proteins having archaeal homologs include four of the five proteins composing yeast U3 snoRNP subcomplex, two of the three proteins composing Mpp10 subcomplex, four of the 20 unclassified nonribosomal proteins, and four of the five ribosomal proteins. Not any homologs to the proteins of the other four subcomplexes of SSU processome were found in archaea. Nine of the 11 archaeal homologs, including fibrillarin, Nop56/58, L7Ae, Rps4e, Utp23/24, Krr1, Rps4, Rps6e, and Rps11, are ubiquitously present in the three investigated phyla of archaea, Crenarchaeota, Euryarchaeota, and Nanoarchaeota, suggesting they at least have already arisen in the last common ancestor of the extant archaea, while the other two of the 11 archaeal homologs, Imp4 and Nep1, are found in all the investigated species of Crenarchaeota and most investigated species of Euryarchaeota but not in Nanoarchaeota (table 2).

**Table 2:** Phylogenetic Distribution of Eukaryotic SSU Processome Protein Homologs in Archaea

	Subcomplex	Protein	Crenarchaeota (18)			Euryarchaeota (48)									
			Desulfurococcales (6)	Sulfolobales (5)	Thermoproteales (7)	Archaeoglobales (3)	Halobacteriales (10)	Methanobacteriale (4)	Methanococcales (8)	Methanomicrobial (5)	Methanopyrales (1)	Methanosarcinale (6)	Thermococcales (7)	Thermoplasmales (3)	Nanoarchaeota (1)
Nonribosomal proteins	U3 snoRNP	Nop1	+	+	+	+	+	+	+	+	+	+	+	+	+
		Nop56	+	+	+	+	+	+	+	+	+	+	+	+	+
		Nop58													
		Snu13	+	+	+	+	+	+	+	+	+	+	+	+	+

	Mpp10	Imp3	+	+	+	+	+	+	+	+	+	+	+	+	+
		Imp4	+	+	+	+	_	+	+	_	+	5 (+)	+	_	_

	Unclassified proteins	Utp23	+	+	+	+	+	+	+	4 (+)	+	+	+	+	+
		Utp24													
		Emg1	+	+	+	+	_	_	5 (+)	_	+	1 (+)	+	+	_
		Krr1	+	+	+	+	+	+	+	+	+	+	+	+	+

Ribosomal proteins		Rps4	+	+	+	+	+	+	+	+	+	+	+	+	+
		Rps6	+	+	+	+	+	+	+	+	+	+	+	+	+
		Rps9	+	+	+	+	+	+	+	+	+	+	+	+	+
		Rps14	+	+	+	+	+	+	+	+	+	+	+	+	+

Note.—“+” represents presence in all species of this clade. “−” represents absence in all species of this clade. *N*(+) represents only present in *N* species and absent in others of a given clade.

No homologs to the 26 probable SSU processome proteins were found in either bacteria or archaea at all.

Put succinctly, bacteria have only three homologs to four of the yeast SSU processome proteins, which are all common ribosomal proteins in prokaryotes. Meanwhile, archaea possess 11 homologs to 14 of the yeast SSU processome proteins, which include both ribosomal and nonribosomal proteins, and contain most components of the U3 snoRNP and Mpp10 subcomplexes as well as several unclassified proteins.

### Functional Domain Composition of Eukaryote-Specific SSU Processome Proteins

Of the 51 confirmed SSU processome proteins, 37 are eukaryote-specific proteins that include 36 nonribosomal proteins and one ribosomal protein. Three (Utp8, Utp9, and Utp16) of the 37 eukaryote-specific proteins are specifically present in Saccharomycotina, and only one of them, Rrp7, is specifically absent in excavates. The other 33 ones are widely distributed. When the 37 eukaryote-specific proteins were subjected to protein domain composition analysis, we found that all the four specifically present/absent proteins exclusively contain eukaryotic protein domains. The domain compositions of the 33 widely distributed proteins are as follows: 1) 14 proteins exclusively contain eukaryotic domains; 2) 11 proteins only contain prokaryote-original domains (five of them comprised only ancient domains, four only bacterial domains, and two both ancient and bacterial domains); 3) eight proteins contain both eukaryotic- and prokaryote-original domains (seven of them contain both eukaryotic and bacterial domains, and the other one contain ancient, archaeal, and eukaryotic domains; table 3).

**Table 3:** Domain Composition of the 37 Eukaryote-Specific SSU Processome Proteins

Protein Name	Domain Composition	Functional Domain in Yeast				Domain in Archaea		Domain in Bacteria			
Rrp5	Ancient	S1(PF00575.17)				S1		S1			
Dbp8	Ancient	DEAD(PF00270.23)	Helicase_C(PF00271.25)			DEAD	Helicase_C	DEAD	Helicase_C		
Rcl1	Ancient	RTC(PF01137.15)	RTC_insert(PF05189.7)			RTC	RTC_insert	RTC	RTC_insert		
Rok1	Ancient	DEAD(PF00270.23)	Helicase_C(PF00271.25)			DEAD	Helicase_C	DEAD	Helicase_C		
Rrp3	Ancient	DEAD(PF00270.23)	Helicase_C(PF00271.25)			DEAD	Helicase_C	DEAD	Helicase_C		
Bms1	Ancient + A + E	GTP_EFTU(PF00009.21)	AARP2CN(PF08142.6)	DUF663(PF04950.6)		GTP_EFTU	DUF663	GTP_EFTU			
Dhr1	Ancient + B	DEAD(PF00270.23)	Helicase_C(PF00271.25)	HA2(PF04408.17)	OB_NTP_bind(PF07717.10)	DEAD	Helicase_C	DEAD	Helicase_C	HA2	OB_NTP_bind
Dhr2	Ancient + B	DEAD(PF00270.23)	Helicase_C(PF00271.25)	HA2(PF04408.17)	OB_NTP_bind(PF07717.10)	DEAD	Helicase_C	DEAD	Helicase_C	HA2	OB_NTP_bind
Rrp9	B	WD40(PF00400.26)						WD40			
Utp4	B	WD40(PF00400.26)						WD40			
Utp17	B	WD40(PF00400.26)						WD40			
Utp18	B	WD40(PF00400.26)						WD40			
Sof1	B + E	WD40(PF00400.26)	Sof1(PF04158.8)					WD40			
Utp1	B + E	WD40(PF00400.26)	Utp12(PF04003.6)					WD40			
Utp7	B + E	WD40(PF00400.26)	BING4CT(PF08149.5)					WD40			
Utp12	B + E	WD40(PF00400.26)	Utp12(PF04003.6)					WD40			
Utp13	B + E	WD40(PF00400.26)	Utp13(PF08625.5)					WD40			
Utp15	B + E	WD40(PF00400.26)	UTP15_C(F09384.4)					WD40			
Utp21	B + E	Utp21(PF04192.6)	WD40(PF00400.26)					WD40			
Mpp10	E	Mpp10(PF04006.6)									
Utp2	E	Nop14(PF04147.6)									
Utp3	E	Sas10_Utp3(PF04000.9)	Sas10_Utp3_C(PF09368.4)								
Utp5	E	Utp12(PF04003.6)									
Utp6	E	U3_assoc_6(PF08640.5)	DUF1740(PF08424.4)								
Utp8	E	Utp8(PF10395.3)									
Utp9	E	Utp12(PF04003.6)									
Utp10	E	U3snoRNP10(PF12397.2)	BP28CT(PF08146.6)								
Utp11	E	Utp11(PF03998.7)									
Utp14	E	Utp14(PF04615.7)									
Utp16	E	U3_snoRNA_assoc(PF08297.5)									
Noc4	E	CBF(PF03914.11)									
Utp20	E	DRIM(PF07539.6)									
Utp22	E	Nrap(PF03813.8)									
Utp25	E	DUF1253(PF06862.6)									
Rps7	E	Ribosomal_S7e(PF01251.12)									
Rrp7	E	RRP7(PF12923.1)									
Rrp36	E	DUF947(PF06102.6)									

Among the 26 probable proteins, 13 contain only eukaryotic domains, 12 contain only prokaryote-original domains, and the other one has no characteristic domain (supplementary data S7, Supplementary Material online).

These protein domain composition analysis results indicate that about half of these eukaryote-specific SSU processome proteins, either confirmed or probable ones, are built up only with eukaryotic domains and the other half are formed largely or only by recruiting prokaryote-original domains. This means that these eukaryote-specific proteins arose after the emergence of eukaryotes through different mechanisms, de novo innovation, recruitment of prokaryote-original domains, or the combination of the two.

### Phylogenetic Correlation of Eukaryotic SSU Processome Proteins with Their Prokaryotic Homologs

From the results of our phylogenetic distribution investigation and protein annotation of the obtained homologs, we found that among the eukaryotic SSU processome proteins there are probably three pairs of paralogs—Nop56 and Nop58; Utp23 and Utp24; Rps9 and Imp3. Likewise, five of the eukaryotic SSU processome proteins, Nop56 and Nop58, Imp4, Krr1, and Snu13, each may have their respective eukaryotic non-SSU processome protein paralogs Prp31, Rpf1, Pno1, and Nhp2. Interestingly, all the nine SSU processome proteins having probable paralogs belong to those eukaryotic SSU processome proteins that have prokaryotic homologs, and on the contrary, none of all the eukaryote-specific SSU processome proteins were found to possess paralogs.

**Fig. 1.—:**
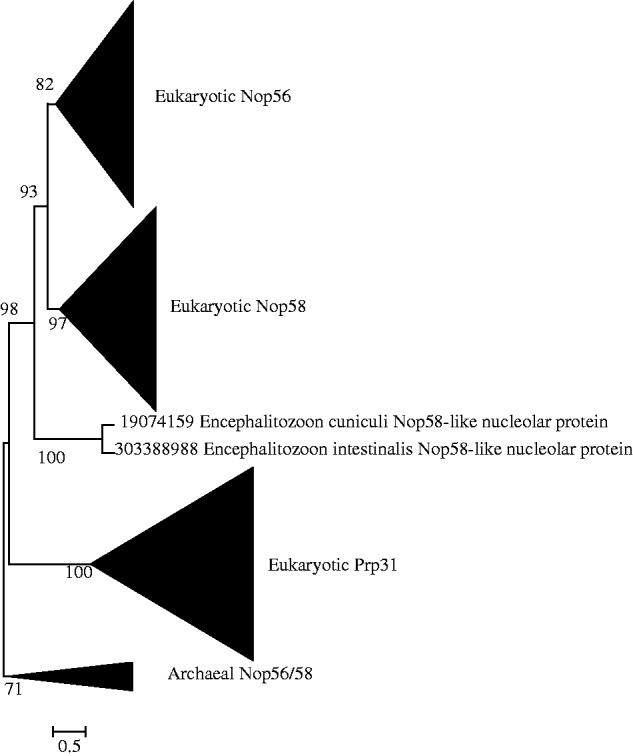
Maximum likelihood phylogenetic tree of the 288 obtained homologous sequences to Nop56 and Nop58. The 390 conserved sites in the alignment were used for the tree construction. Numbers at branches represent bootstrap values. The scale bar represents the average number of substitutions per site.

**Fig. 2.—:**
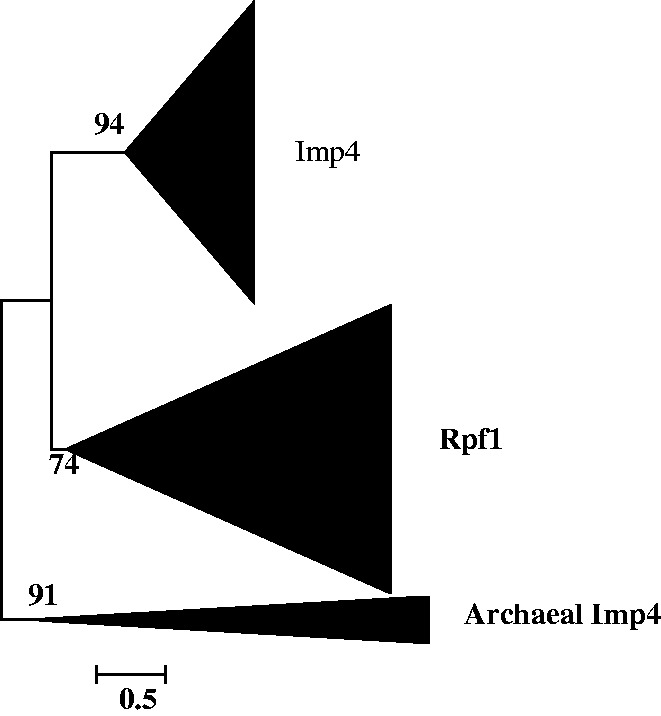
Maximum likelihood phylogenetic tree of the 165 obtained homologous sequences to Imp4. The 374 conserved sites in the alignment were used for the tree construction. Numbers at branches represent bootstrap values. The scale bar represents the average number of substitutions per site.

**Fig. 3.—:**
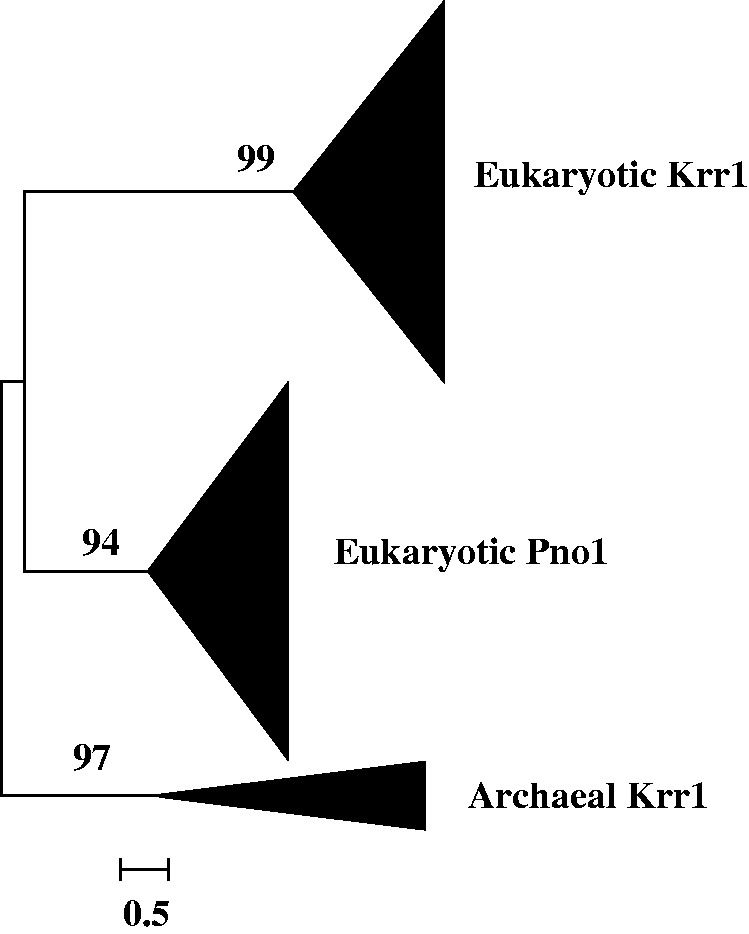
Maximum likelihood phylogenetic tree of the 171 obtained homologous sequences to Krr1. The 450 conserved sites in the alignment were used for the tree construction. Numbers at branches represent bootstrap values. The scale bar represents the average number of substitutions per site.

**Fig. 4.—:**
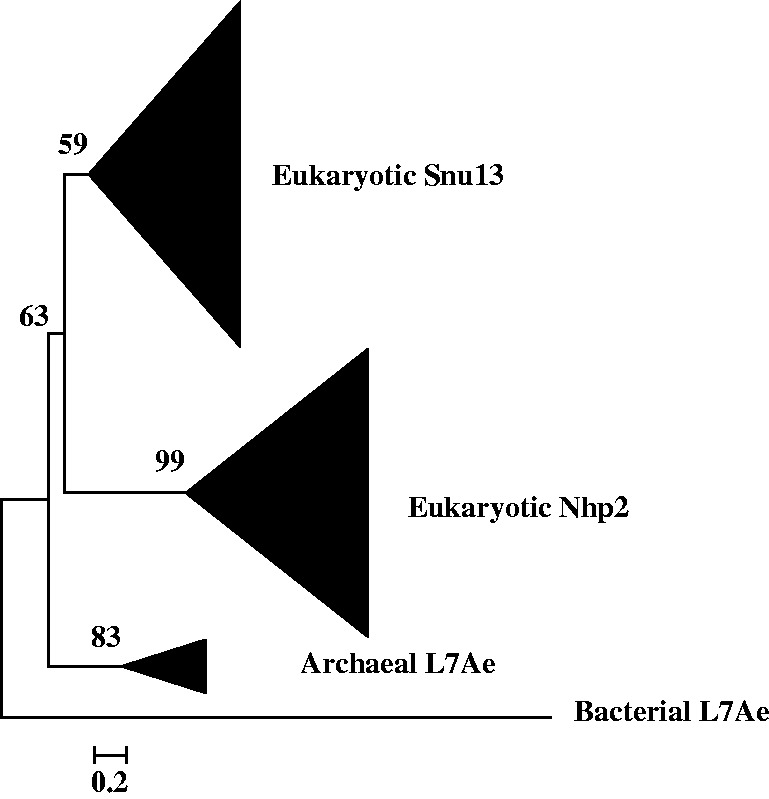
Maximum likelihood phylogenetic tree of the 182 obtained homologous sequences to Snu13. The 131 conserved sites in the alignment were used for the tree construction. Numbers at branches represent bootstrap values. The scale bar represents the average number of substitutions per site.

To investigate the prokaryotic origin of eukaryotic SSU processome proteins and the evolution of them in eukaryotes, we subjected all the 14 eukaryotic SSU processome proteins that have prokaryotic homologs to single-gene phylogenetic analysis and reconstruct 11 phylogenetic trees as three pairs (mentioned earlier) of them were paralogs reciprocally. Fasttree and PhyML programs produced similar trees with minor differences in topologies, and thus the Fasttree trees are displayed here as representatives. Of the 11 phylogenetic trees, eight (Nop56/Nop58, Imp4, Krr1, Nop1, Utp23 and Utp24, Emg1, Rps4, and Rps6 trees) contain only archaeal homologs (figs. 1–3, supplementary data S8, Supplementary Material online), while the other three (Snu13, Imp3/Rps9, and Rps14 trees) contain both archaeal and bacterial homologs, but in each of them the archaeal homolog clade is more closely related to the eukaryotic clade(s) than the bacterial homolog clade is (fig. 4, supplementary data S9, Supplementary Material online). Altogether, these findings suggest that eukaryotes must have vertically inherited these 11 protein genes from archaea during the origin of eukaryotes from prokaryotes, and no bacterial protein genes contributed directly to the origin of eukaryotic SSU processome at all. Generally, all the archaeal homologs were clustered into a monophyletic clade as a sister group to the eukaryotic protein clade(s) on each tree, indicating that the genes of these eukaryotic SSU processome proteins, including the four non-SSU processome protein paralogs: Prp31, Rpf1, Pno1, and Nhp2, each originate from the common ancestor of archaea. However, there are three trees (fig. 2, supplementary data S8*a* and S9*b*, Supplementary Material online), on which archaeal homologs were not clustered into one monophyletic clade, but only two of them form a sister group to the eukaryotic clade(s), possibly as a result of insufficient phylogenetic signal on these three protein sequences to accurately portray the real relationship between these archaeal and eukaryotic proteins due to sequence composition.

On the six trees that contain paralogs, we found two particular kinds of topology: 1) on the Nop56/Nop58, Utp23/Utp24, Imp3/Rps9 trees, one pair of paralogs of eukaryotic SSU processome proteins were included in and recovered to be two separate clades on each of the trees (fig. 1, supplementary data S8*b* and S9*a*, Supplementary Material online), suggesting that the three pairs of SSU processome proteins each were produced through ancient gene duplication of a common archaeal ancestral protein and then functional divergence into two different SSU processome proteins; 2) on the Nop56/Nop58 tree and the trees of three other proteins—Imp4, Krr1, Snu13, the eukaryotic non-SSU processome proteins were recovered to be a sister clade to their eukaryotic SSU processome paralog clade (figs. 2–4), suggesting that these paralog pairs were each produced through ancient gene duplication but with one of the two copies becoming a non-SSU processome protein. In addition, on each of the 11 single-gene phylogenetic trees, the multiple copies of some SSU processome proteins in various species (e.g., *Arabidopsis thaliana*, *T. vaginalis*, *P. tetraurelia*, and so on) were generally clustered together first with high support values, respectively (figs. 1–4, supplementary data S8 and S9, Supplementary Material online), suggesting these multiple-copied proteins must be produced through relatively recent species-specific gene duplication. For Nop1, besides such species-specific gene duplication was found in many individual species of Kinetoplastid, a lineage-specific gene duplication in this lineage was also found (supplementary data S10, Supplementary Material online). Therefore, in the evolution of SSU processome in eukaryotes, most of its component protein genes that were inherited from archaea underwent gene duplication to enlarge the components of SSU processome and to produce some non-SSU processome proteins. This is especially true for the protein Nop1 (supplementary data S10, Supplementary Material online): originating from archaeal fibrillarin and undergoing a series of gene duplications during the diversification of eukaryotes.

## Discussion

A principal challenge in dealing with the origin of eukaryotic cells is to understand the origin of eukaryotic cell structures and eukaryote-specific cellular processes in the onset of the appearance of the last eukaryotic common ancestor (LECA). The presence of the eukaryotic multiprotein complexes (EMC) involved in various cellular processes or structures is one of the main distinctive differences between eukaryotic and prokaryotic cells. As a kind of EMC, SSU processome has not been studied widely and deeply to date. In the present study, however, we not only proved the SSU processome to be a ubiquitous mechanism in eukaryotes but also explored its origin and evolution in eukaryotes and throw light on the origin of the nucleolus.

### SSU Processome’s Emergence as a Fairly Complete EMC in the LECA

**Fig. 5.—:**
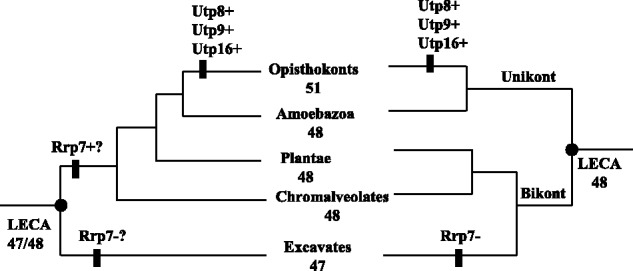
SSU processome components traceable to the LECA. SSU processome composition in the LECA based on two alternative roots of the eukaryote tree. In the left-hand tree, excavates are the outgroup. The right-hand tree is rooted on the basis of the unikont/bikont bifurcation. Gains (+) and losses (−) in different lineages are indicated under each scenario. Where gains and losses are equally probable, these are marked by (?).

As the only RNA component of SSU processome known up to now, U3 snoRNA shows an evolutionarily conserved presence in eukaryotes (Marz and Stadler 2009). Our investigation indicated that in actuality most of the eukaryotic SSU processome protein components are also widely present in the five major eukaryotic supergroups—except three Saccharomycotina-specific proteins and one probably excavate-specifically absent protein, all the other 47 confirmed yeast SSU processome proteins and most of the 26 probable proteins are ubiquitous in all the five eukaryotic supergroups. Accordingly, both the U3 snoRNA and the majority of the SSU processome proteins can be traced back to LECA. Generally, the ability to assign a complex to LECA differs depending on the topology of the eukaryote tree. According to the two different hypothetical eukaryote trees (Stechmann and Cavalier-Smith 2003, 2002; Morrison et al. 2007), 47 or 48 of the confirmed yeast SSU processome proteins can be thought to be present in LECA (fig. 5). The only equivocal component is Rrp7, because it might arose after the branching off of excavates from the eukaryotic trunk when excavates are considered to be basal on the eukaryote tree (Morrison et al. 2007) or might also be specifically lost in excavates if the root of the eukaryote tree is placed between unikonts and bikonts (Stechmann and Cavalier-Smith 2002, 2003; Roger and Simpon 2009; Koonin 2010; Katz 2012). According to the currently popular concept that most excavates branched at the bottom of the eukaryotic tree is a long-branch attraction artifact in phylogenetic tree reconstructions (Derelle and Lang 2012), we claim that Rrp7 is more likely present in the LECA and was then lost in excavates. Anyhow, our results indicate that the majority of eukaryotic SSU processome components arose in the forming of LECA and thus a fairly complete modern-like SSU processome must therefore have finally emerged in the LECA. This is consistent with the previous study, which showed that numerous eukaryotic paralogs of superstructure-forming proteins, including many WD 40 domain containing proteins, are present in the LECA (Makarova et al. 2005).

### Prokaryotic Origin of Eukaryotic SSU Processome and Then Its Evolution in the Divergence of Eukaryotes

Because the LECA harbored SSU processome, how prokaryotes contributed to the origin of such a eukaryotic complex and how this complex evolved during the divergence of eukaryotes are interesting lines of inquiry. Except for the four proteins whose bacterial homologs all are common ribosomal proteins in prokaryotes, no other SSU processome proteins were found to possess bacterial homologs. This is consistent with the fact that no traceable evidence for any homologs to eukaryotic SSU processome proteins or similar pre-rRNA processing mechanism has ever been reported in bacteria so far. In addition, it was once reported that the eukaryotic proteins involving information-processing systems are of archaeal origin (Yutin et al. 2008). Thus, our work further confirms that the SSU processome did not arise in bacteria at all.

Besides the seven previously reported archaeal homologs to SSU processome nonribosomal proteins (Mayer et al. 2001; Eschrich et al. 2002; Kuhn et al. 2002), we found three more such kind of protein homologs in archaea, and we also found that four of the five eukaryotic SSU processome ribosomal proteins have homologs in many more archaea. Thus, altogether, at least 14 proteins of the eukaryotic SSU processome have been found to possess 11 homologs in archaea. Most of these homologs are present in almost all the investigated species of the three archaeal phyla, except two of them seem to be absent in the sole investigated species of Nanoarchaeota and in a few species of Euryarchaeota. Therefore, at least nine SSU processome protein homologs might have already arisen in the last common ancestor of the extant archaea. Among them, the four archaeal homologs to the eukaryotic protein components of the U3 snoRNP subcomplex and the two to those of the Mpp10 subcomplex all possess the conserved functional domains as their eukaryotic counterparts do (supplementary data S11, Supplementary Material online). Therefore, these archaeal homologs are probably able to constitute the two subcomplexes involving the archaeal pre-rRNA processing, though in a relatively simple form. In *S. cerevisiae*, the formation of five short duplexes between the U3 snoRNA and the 18S pre-rRNA is strictly required for the endonucleolytic cleavages at the A0, A1, and A2 sites, and the expected role of the U3-pre-rRNA duplexes is to guide the SSU processome proteins to the cleavage sites (Beltrame and Tollervey 1995; Sharma and Tollervey 1999; Dutca et al. 2011), indicating the presence of the U3 snoRNP subcomplex is a prerequisite for the formation of SSU processome. Moreover, previous studies indicated that the two proteins, Imp3 and Imp4, are essential to mediate the U3-pre-rRNA interactions and greatly increase the stability of the unstable U3-pre-rRNA duplexes (Gerczei and Correll 2004; Gerczei et al. 2009). Accordingly, the two subcomplexes, U3 snoRNP and Mpp10, are two basic functional subcomplexes of SSU processome, and might be present in archaea. A U3-like snoRNA was once reported to be present in archaea, and a U3-like snoRNP, which contains the U3-like snoRNA and five or six proteins (one of which was reported to have cross reacts with human antifibrillarin antibody), was even reported to mediate SSU pre-rRNA processing in *S. acidocaldarius* (Potter et al. 1995; Omer et al. 2000), but the identification of U3-like snoRNA in archaea was subsequently determined to be an error (Russell et al. 1997), and up to date, there is no data supporting the existence of U3 snoRNA in archaea. However, it is still possible that related snoRNAs are involved in the processing of archaeal pre-rRNA (Brown and Doolittle 1997), because rRNA processing similar to that of eukaryotes was found in archaea (Durovic and Dennis 1994), and many homologs of snoRNAs were found in archaea (Omer et al. 2000). Certainly, the identification of a small RNA functioning as U3 snoRNA is awaited.

Taken together with our earlier results, it seems plausible that a probable rudimentary but functional SSU pre-rRNA processing complex, similar to eukaryotic SSU processome but with fewer components, probably have already arisen in archaea. But this must await further experimental evidence.

As mentioned earlier, at least the 47 confirmed SSU processome proteins are traceable to LECA. This means that on the basis of archaeal homologous proteins (including 11 proteins), at least 36 novel confirmed SSU processome proteins must have arisen in the formation of LECA. Our phylogenetic analysis showed that 3 of the 36 proteins derived from ancient gene duplication of their archaeal ancestral proteins. Our protein domain composition analysis indicated that the other 33 are nearly equally built up with only eukaryotic protein domains or with both eukaryotic protein domains and preexisting prokaryote-original protein domains (jointly including ancient, bacterial, and archaeal protein domains) together, implying that besides the de novo innovation, fusion of diverse building blocks of prokaryotic and eukaryotic origins is another important mechanism for the emergence of these novel proteins, which seems to follow the same general principle that governs evolution of other eukaryotic functional systems, such as RNA interference system or the spliceosome (Collins and Penny 2005; Shabalina and Koonin 2008).

The eukaryotic processome has a sheer size in contrast to its potential archaeal counterpart. Three main factors may relate to this: 1) for bioenergetic reasons, eukaryotes are able to expand genome sizes over several orders of magnitude and hence are under much less selection for streamlining than prokaryotes, giving them the scope to “experiment” with gene duplications and protein sequence space (Lane and Martin 2010); 2) even unicellular eukaryotes have relatively small population sizes compared with prokaryotes, which lowers the strength of selection and tends to favor molecular ratchets producing larger molecular machines than found in prokaryotes (Gray et al. 2010; Lynch 2012); 3) the eukaryotic pre-rRNAs or rRNAs are larger than their bacterial or archaeal counterparts and may thus require additional factors as scaffolds, assembly factors, and regulatory components.

After the emergence of the eukaryotic SSU processome in LECA, we found that the complex also underwent divergences in different lineages and even different species during the divergence of the eukaryotes. First, lineage-specific and species-specific gene duplications occurred frequently. Second, lineage-specific gene gains that increased the complexity of SSU processome were also observed; for example, Saccharomycotina gained some specific proteins. Lastly, there are numerous instances where lineage- and species-specific gene losses also enriched the divergence history of SSU processome in eukaryotes; for example, many components were totally lost in the lineage Microsporidia, and various components of SSU processome were lost in the species *G. lamblia*, and species-specific gene loss is very obvious in several species of Plasmodium and Cryptosporidium. All these lines of evidence suggest the evolutionary plasticity of SSU processome complex in different lineages or even species, though the complex is thought to be a conserved functional one in eukaryotes.

### Implications for the Origin of the Nucleolus and Eukaryotes

The nucleolus is one of the defining features of eukaryotes. It is the site for synthesizing and processing of pre-rRNA in eukaryotes, though without this structure, prokaryotes can also perform these processes in the cytoplasm. Logically, studying the origin of eukaryotic SSU processome would be helpful in understanding the origin of the nucleolus, and by extension, even the eukaryotic cell, in the evolutionary origin of eukaryotes from prokaryotes. Unfortunately, the origin of the nucleolus remains a huge enigma and major challenge for evolutionary biology. By investigating the phylogenetic distribution of human nucleolar protein domains in the three domains of life, Staub et al*.* (2004) proposed the chimeric origin of the nucleolus and considered that the core nucleolar proteins involved in ribosomal assembly originated from an archaeal ancestor, and later, bacterial nucleolar protein domains were added for the evolution of the nucleolus. By the similar analysis of domain composition of protein, our work also showed that, of the eukaryotic SSU processome proteins, many of the part that arose after the emergence of eukaryotes (i.e., many of those that have no prokaryotic homologs) originated by recruiting archaeal-origin domains, bacterial-origin domains, or both. This is consistent with the results of the whole nucleolar proteins of Staub et al. However, when we carried out homolog search and phylogenetic analysis based on the full-length proteins (not just the domains), the results showed that about a quarter of the confirmed eukaryotic SSU processome proteins, which belong to the other part of the eukaryotic SSU processome proteins that have prokaryotic homologs (i.e., those that are considered to be prokaryotic origin), originated from archaea through direct vertical inheritance without any from bacteria. Therefore, the origins of the single molecular machine of the nucleolus—the eukaryotic SSU processome, are revealed more clearly in the present work.

It was proposed that the spread of type II introns and corresponding spliceosomes is adduced as the selective pressure that forged nucleus–cytosol compartmentalization (Martin and Koonin 2006). Further, the emergence of the nucleolus should be tightly related to the subnuclear compartmentalization of the transcription and processing of rRNA in eukaryotic cells. Therefore, the origin of eukaryote-specific nucleolar proteins specifically for the transcription and processing of rRNA should be associated with the evolutionary emergence of the nucleolar structure. We noticed the origin of such specific proteins for the processing of rRNA through gene duplication during the present study. In eukaryotes, Nop56 and Nop58 are involved in pre-rRNA processing, while Prp31 is required for pre-mRNA splicing (Makarova et al. 2002). Obviously, these two kinds of processing of the two different pre-RNAs occur in different regions of the eukaryotic nucleus, which is a typical functional compartmentalization. Archaeal Nop56/58 is involved in both pre-mRNA splicing and pre-rRNA processing and both occur in the cytoplasm (Oruganti et al. 2007; Anderson et al. 2011). Our phylogenetic tree showed that Nop56 and Nop58 clustered together first, then Prp31 formed a sister group to them, and archaeal Nop56/58 formed an outgroup to them all, indicating that the archaeal ancestral protein Nop56/58 finally underwent ancient gene duplication and functional divergence two times to produce eukaryotic Prp31, Nop56, and Nop58 in the LECA (fig. 1). Such gene duplication and functional divergence must have contributed to the compartmentalization of two different kinds of pre-RNA processing, which is in turn related to the evolutionary emergence of the nucleolus.

Another five ancient gene duplications that also produced eukaryotic paralogs were found in the course of this study (figs. 2–4, supplementary data S8*b* and S9*a*, Supplementary Material online). Among these produced paralogs, besides the eukaryotic SSU processome proteins, the other proteins Rpf1, Pno1, and Nhp2 (figs. 2–4) are non-SSU processome proteins but nucleolar proteins are required for another function—ribosome biogenesis in eukaryotes. Such functional specialization facilitated by gene duplication is very common in eukaryotes; for example, ancestral RNA polymerase experienced duplications and diverged into three functionally distinct RNA polymerase I, II, and III to transcribe rRNA, mRNA, and tRNA, respectively (Archambault and Friesen 1993). Ancient gene duplication has been considered to play pivotal roles in the emergence of the eukaryotic cell complexity (Makarova et al. 2005). We speculate that the gene duplication of some of the SSU processome proteins might make contribution to the emergence of the nucleolus in the LECA.

Phylogenomic reconstruction of eukaryote supergroups suggested that LECA was already a highly complex and modern-like eukaryotic cell (Koonin 2010). In the present work, we showed that a fairly complex SSU processome was already present in the LECA and further implied that the LECA might even have harbored a nucleolus.

## Supplementary Material

Supplementary data S1–S11 and figures S1–S4 are available at *Genome Biology and Evolution* online (http://www.gbe.oxfordjournals.org/).

Supplementary Data
