# Supplementary material for: Origin and Evolution of the Eukaryotic SSU Processome Revealed by a Comprehensive Genomic Analysis and Implications for the Origin of the Nucleolus
Source: Genome Biol Evol. 2013 Nov 7;5(12):2255–67. doi: 10.1093/gbe/evt173 (PMC3879963; doi:10.1093/gbe/evt173)
Supplement: Supplementary Data [file supp_evt173_suppl_data.zip › Supplementary_data_1.doc]

**Supplementary_data 1. Eukaryotic organisms used in this study**.

| **Organisms** | **Code** | **Lineage** | **Supergroups** | **Data Source** |
| --- | --- | --- | --- | --- |
| ***Homo sapiens*** | **Hsa** | **Vertebrates** | **Opisthokonts** | **NCBI RefSeq** |
| ***Mus musculus*** | **Mmu** | **Vertebrates** | **Opisthokonts** | **NCBI RefSeq** |
| ***Rattus norvegicus*** | **Rno** | **Vertebrates** | **Opisthokonts** | **NCBI RefSeq** |
| ***Gallus gallus*** | **Gga** | **Vertebrates** | **Opisthokonts** | **NCBI RefSeq** |
|  |  |  |  |  |
| ***Xenopus tropicalis*** | **Xtr** | **Vertebrates** | **Opisthokonts** | **NCBI RefSeq** |
| ***Danio rerio*** | **Dre** | **Vertebrates** | **Opisthokonts** | **NCBI RefSeq** |
| ***Ciona intestinalis*** | **Cin** | **Tunicates** | **Opisthokonts** | **NCBI RefSeq** |
| ***Strongylocentrotus purpuratus*** | **Spu** | **Echinodermatas** | **Opisthokonts** | **NCBI RefSeq** |
| ***Anopheles gambiae*** | **Aga** | **Dipterans** | **Opisthokonts** | **NCBI RefSeq** |
| ***Apis mellifera*** | **Ame** | **Dipterans** | **Opisthokonts** | **NCBI RefSeq** |
| ***Drosophila melanogaster*** | **Dme** | **Dipterans** | **Opisthokonts** | **NCBI RefSeq** |
| ***Drosophila yakuba*** | **Dya** | **Dipterans** | **Opisthokonts** | **NCBI RefSeq** |
| ***Caenorhabditis elegans*** | **Cel** | **Nematodes** | **Opisthokonts** | **NCBI RefSeq** |
| ***Caenorhabditis briggsae*** | **Cbr** | **Nematodes** | **Opisthokonts** | **NCBI RefSeq** |
| ***Schistosoma mansoni*** | **Sma** | **Platyhelminthes** | **Opisthokonts** | **NCBI RefSeq** |
|  |  |  |  |  |
| ***Pichia pastoris*** | **Ppa** | **Ascomycetes; Saccharomycotina** | **Opisthokonts** | **NCBI RefSeq** |
| ***Ashbya gossypii*** | **Ago** | **Ascomycetes; Saccharomycotina** | **Opisthokonts** | **NCBI RefSeq** |
| ***Kluyveromyces lactis*** | **Kla** | **Ascomycetes; Saccharomycotina** | **Opisthokonts** | **NCBI RefSeq** |
| ***Candida dubliniensis*** | **Cdu** | **Ascomycetes; Saccharomycotina** | **Opisthokonts** | **NCBI RefSeq** |
| ***Candida glabrata***  ***CCMP1335*** | **Cgl** | **Ascomycetes; Saccharomycotina** | **Opisthokonts** | **NCBI RefSeq** |
| ***Candida albicans***  ***CCAP 1055/1*** | **Cal** | **Ascomycetes; Saccharomycotina** | **Opisthokonts** | **NCBI RefSeq** |
| ***Debaromyces hansenii*** | **Dha** | **Ascomycetes; Saccharomycotina** | **Opisthokonts** | **NCBI RefSeq** |
| ***Scheffersomyces stipitis*** | **Sst** | **Ascomycetes; Saccharomycotina** | **Opisthokonts** | **NCBI RefSeq** |
| ***Zygosaccharomyces rouxii*** | **Zro** | **Ascomycetes; Saccharomycotina** | **Opisthokonts** | **NCBI RefSeq** |
| ***Vanderwaltozyma polyspora*** | **Vpo** | **Ascomycetes; Saccharomycotina** | **Opisthokonts** | **NCBI RefSeq** |
| ***Lachancea thermotolerans***  ***MHOM/BR/75/M2904*** | **Lth** | **Ascomycetes; Saccharomycotina** | **Opisthokonts** | **NCBI RefSeq** |
| ***Yarrowia lipolytica*** | **Yli** | **Ascomycetes; Saccharomycotina** | **Opisthokonts** | **NCBI RefSeq** |
| ***Neurospora crassa*** | **Ncr** | **Ascomycetes; Pezizomycotina** | **Opisthokonts** | **NCBI RefSeq** |
| ***Podospora anserina*** | **Pan** | **Ascomycetes; Pezizomycotina** | **Opisthokonts** | **NCBI RefSeq** |
| ***Phaeosphaeria nodorum*** | **Pno** | **Ascomycetes; Pezizomycotina** | **Opisthokonts** | **NCBI RefSeq** |
| ***Penicillium chrysogenum*** | **Pch** | **Ascomycetes; Pezizomycotina** | **Opisthokonts** | **NCBI RefSeq** |
| ***Magnaporthe oryzae*** | **Mor** | **Ascomycetes; Pezizomycotina** | **Opisthokonts** | **NCBI RefSeq** |
| ***Chaetomium globosum*** | **Cglo** | **Ascomycetes; Pezizomycotina** | **Opisthokonts** | **NCBI RefSeq** |
| ***Aspergillus terreus*** | **Ate** | **Ascomycetes; Pezizomycotina** | **Opisthokonts** | **NCBI RefSeq** |
| ***Aspergillus nidulans*** | **Ani** | **Ascomycetes; Pezizomycotina** | **Opisthokonts** | **NCBI RefSeq** |
| ***Schizosaccharomyces pombe*** | **Spo** | **Ascomycetes; Schizosaccharomycetes** | **Opisthokonts** | **NCBI RefSeq** |
| ***Ustilago maydis*** | **Uma** | **Basidiomycete** | **Opisthokonts** | **NCBI RefSeq** |
| ***Encephalitozoon cuniculi*** | **Ecu** | **Microsporidia** | **Opisthokonts** | **EuPathDB** |
| ***Encephalitozoon intestinalis*** | **Ein** | **Microsporidia** | **Opisthokonts** | **EuPathDB** |
| ***Monosiga brevicollis*** | **Mbr** | **Choanoflagellates** | **Opisthokonts** | **Joint Genome Institute** |
| ***Entamoeba histolytica*** | **Ehi** | **Entamoebida** | **Amoebozoa** | **EuPathDB** |
| ***Dictyostelium discoideum*** | **Ddi** | **Mycetozoa** | **Amoebozoa** | **NCBI RefSeq** |
| ***Arabidopsis thaliana*** | **Ath** | **Streptophytes** | **Plantae** | **NCBI RefSeq** |
| ***Oryza sativa*** | **Osa** | **Streptophytes** | **Plantae** | **NCBI RefSeq** |
| ***Physcomitrella patens*** | **Ppat** | **Streptophytes** | **Plantae** | **Joint Genome Institute** |
| ***Populus trichocarpa*** | **Ptr** | **Streptophytes** | **Plantae** | **Joint Genome Institute** |
| ***Chlamydomonas reinhardtii*** | **Cre** | **Chlorophytes** | **Plantae** | **Joint Genome Institute** |
| ***Volvox carteri*** | **Vca** | **Chlorophytes** | **Plantae** | **Joint Genome Institute** |
| ***Ostreococcus lucimarinus*** | **Olu** | **Chlorophytes** | **Plantae** | **Joint Genome Institute** |
| ***Cyanidioschyzon merolae*** | **Cme** | **Rhodophyte** | **Plantae** | **U. of Tokyo** |
| ***Trichomonas vaginalis*** | **Tva** | **Parabasalids** | **Excavates** | **EuPathDB** |
| ***Trypanosoma brucei*** | **Tbr** | **Kinetoplastids** | **Excavates** | **EuPathDB** |
| ***Trypanosoma cruzi*** | **Tcr** | **Kinetoplastids** | **Excavates** | **EuPathDB** |
| ***Leishmania major*** | **Lma** | **Kinetoplastids** | **Excavates** | **EuPathDB** |
| ***Leishmania infantum*** | **Lin** | **Kinetoplastids** | **Excavates** | **EuPathDB** |
| ***Leishmania braziliensis*** | **Lbr** | **Kinetoplastids** | **Excavates** | **EuPathDB** |
| ***Giardia lamblia*** | **Gla** | **Diplomonads** | **Excavates** | **EuPathDB** |
| ***Naegleria gruberi*** | **Ngr** | **Heterolobosea** | **Excavates** | **Joint Genome Institute** |
| ***Tetrahymena thermophila*** | **Tth** | **Ciliate** | **Chromalveolates** | **NCBI RefSeq** |
| ***Paramecium tetraurelia*** | **Pte** | **Ciliate** | **Chromalveolates** | **NCBI RefSeq** |
| ***Plasmodium berghei*** | **Pbe** | **Apicomplexans** | **Chromalveolates** | **NCBI RefSeq** |
| ***Plasmodium falciparum*** | **Pfa** | **Apicomplexans** | **Chromalveolates** | **NCBI RefSeq** |
| ***Plasmodium knowlesi*** | **Pkn** | **Apicomplexans** | **Chromalveolates** | **NCBI RefSeq** |
| ***Plasmodium vivax*** | **Pvi** | **Apicomplexans** | **Chromalveolates** | **NCBI RefSeq** |
| ***Plasmodium yoelii*** | **Pyo** | **Apicomplexans** | **Chromalveolates** | **NCBI RefSeq** |
| ***Toxoplasma gondii*** | **Tgo** | **Apicomplexans** | **Chromalveolates** | **NCBI RefSeq** |
| ***Theileria parva*** | **Tpa** | **Apicomplexans** | **Chromalveolates** | **NCBI RefSeq** |
| ***Cryptosporidium hominis*** | **Cho** | **Apicomplexans** | **Chromalveolates** | **NCBI RefSeq** |
| ***Cryptosporidium parvum*** | **Cpa** | **Apicomplexans** | **Chromalveolates** | **NCBI RefSeq** |
| ***Theileria annulata*** | **Tan** | **Apicomplexans** | **Chromalveolates** | **NCBI RefSeq** |
| ***Babesia bovis*** | **Bbo** | **Apicomplexans** | **Chromalveolates** | **NCBI RefSeq** |
| ***Phytophthora infestans*** | **Pin** | **Heterokonts** | **Chromalveolates** | **Broad Institute** |
| ***Phytophthora sojae*** | **Pso** | **Heterokonts** | **Chromalveolates** | **Joint Genome Institute** |
| ***Phytophthora ramorum*** | **Pra** | **Heterokonts** | **Chromalveolates** | **Joint Genome Institute** |
| ***Thalassiosira pseudonana*** | **Tps** | **Heterokonts** | **Chromalveolates** | **Joint Genome Institute** |
| ***Phaeodactylum tricornutum*** | **Ptri** | **Heterokonts** | **Chromalveolates** | **Joint Genome Institute** |
| ***Ectocarpus siliculosus*** | **Esi** | **Heterokonts** | **Chromalveolates** | **NCBI nr** |
