# Supplementary material for: Origin and Evolution of the Eukaryotic SSU Processome Revealed by a Comprehensive Genomic Analysis and Implications for the Origin of the Nucleolus
Source: Genome Biol Evol. 2013 Nov 7;5(12):2255–67. doi: 10.1093/gbe/evt173 (PMC3879963; doi:10.1093/gbe/evt173)
Supplement: Supplementary Data [file supp_evt173_suppl_data.zip › Supplementary_data_3.doc]

**Supplementary_data 3. Summary of the distributions of the 26 probable proteins in the five eukaryotic supergroups.**

| **Protein name** | **Opisthokonts** | **Amoebozoa** | **Plantae** | **Excavates** | **Chromalveolates** | **LECA** |
| --- | --- | --- | --- | --- | --- | --- |
| **Pol5** | **+** | **+** | **+** | **-**  **-** | **+** | **-** |
| **Cka1** | **+** | **+** | **+** | **+** | **+** | **+** |
| **Cka2** | **+** | **+** | **+** | **+** | **+** | **+** |
| **Ckb1** | **+** | **+** | **+** | **+** | **+** | **+** |
| **Ckb2** | **+** | **+** | **+** | **+** | **+** | **+** |
| **Utp30** | **+** | - | **+** | **+** | **+** | - |
| **Dbp4** | **+** | **+** | **+** | **+** | **+** | **+** |
| **Enp1** | **+** | **+** | **+** | **+** | **+** | **+** |
| **Esf1** | **+** | **+** | **+** | **+** | **+** | **+** |
| **Esf2** | **+** | **+** | **+** | **+** | **+** | **+** |
| **Fal1** | **+** | **+** | **+** | **+** | **+** | **+** |
| **Fyv7** | **+** | - | **+** | - | **+** | - |
| **Gno1** | **+** | - | **+** | **+** | **+** | - |
| **Has1** | **+** | **+** | **+** | **+** | **+** | **+** |
| **Kre33** | **+** | **+** | **+** | **+** | **+** | **+** |
| **Lcp5** | **+** | **+** | **+** | **+** | **+** | **+** |
| **Ltv1** | **+** | - | - | **+** | - | - |
| **Mrd1** | **+** | **+** | **+** | **+** | **+** | **+** |
| **Nop9** | **+** | **+** | **+** | **+** | **+** | **+** |
| **Nsr1** | **+** | **-** | **+** | **+** | **+** | **-** |
| **Pfa1** | **+** | **-** | **+** | **+** | **+** | **-** |
| **Prp43** | **+** | **+** | **+** | **+** | **+** | **+** |
| **Sen1** | **+** | **+** | **+** | **+** | **+** | **+** |
| **Sgd1** | **+** | **+** | **+** | **+** | **+** | **+** |
| **Slx9** | **+** | - | - | - | - | - |
| **YGR251W** | **+** | - | - | - | - | - |

Note: “+ ” represents presence; “-” represents absence.
