# Supplementary material for: Origin and Evolution of the Eukaryotic SSU Processome Revealed by a Comprehensive Genomic Analysis and Implications for the Origin of the Nucleolus
Source: Genome Biol Evol. 2013 Nov 7;5(12):2255–67. doi: 10.1093/gbe/evt173 (PMC3879963; doi:10.1093/gbe/evt173)
Supplement: Supplementary Data [file supp_evt173_suppl_data.zip › Supplementary_data_5.doc]

**Supplementary_data 5. Bacterial homologous proteins to the yeast SSU processome proteins and their functions.**

| **Protein** | **Bacterial homolog** | **Function** |
| --- | --- | --- |
| Snu13 | L7Ae | Large ribosomal protein |
| Imp3 | Rps4e | Small ribosomal protein |
| Rps9 |  |  |
| Rps14 | Rps11 |  |
