# Supplementary material for: Origin and Evolution of the Eukaryotic SSU Processome Revealed by a Comprehensive Genomic Analysis and Implications for the Origin of the Nucleolus
Source: Genome Biol Evol. 2013 Nov 7;5(12):2255–67. doi: 10.1093/gbe/evt173 (PMC3879963; doi:10.1093/gbe/evt173)
Supplement: Supplementary Data [file supp_evt173_suppl_data.zip › Supplementary_data_6.doc]

**Supplementary_data 6. The distribution of SSU Processome proteins in the archaea investigated.**

|  |  | **Species** | **Nop1** | **Nop56/58** | **Snu13** | **Imp3/Rps9** | **Imp4** | **Utp23/24** | **Emg1** | **Krr1** | **Rps4** | **Rps6** | **Rps14** |
| --- | --- | --- | --- | --- | --- | --- | --- | --- | --- | --- | --- | --- | --- |
| **Crenarchaeota** | **Desulfurococcales**  **（6）** | ***Aeropyrum pernix K1*** | **+** | **+** | **+** | **+** | **+** | **+** | **+** | **+** | **+** | **+** | **+** |
|  |  | ***Desulfurococcus kamchatkensis 1221n*** | **+** | **+** | **+** | **^+^** | **+** | **+** | **+** | **+** | **+** | **+** | **+** |
|  |  | ***Hyperthermus butylicus DSM 5456*** | **+** | **+** | **+** | **+** | **+** | **+** | **+** | **+** | **+** | **+** | **+** |
|  |  | ***Ignicoccus hospitalis KIN4/I*** | **+** | **+** | **+** | **+** | **+** | **+** | **+** | **+** | **+** | **+** | **+** |
|  |  | ***Staphylothermus marinus F1*** | **+** | **+** | **+** | **+** | **+** | **+** | **+** | **+** | **+** | **+** | **+** |
|  |  | ***Thermosphaera aggregans DSM 11486*** | **+** | **+** | **+** | **+** | **+** | **-** | **+** | **+** | **+** | **+** | **+** |
|  | **Sulfolobales （5）** | ***Metallosphaera sedula DSM 5348*** | **+** | **+** | **+** | **+** | **+** | **-** | **+** | **+** | **+** | **+** | **+** |
|  |  | ***Sulfolobus acidocaldarius DSM 639*** | **+** | **+** | **+** | **+** | **+** | **+** | **+** | **+** | **+** | **+** | **+** |
|  |  | ***Sulfolobus islandicus*** | **+** | **+** | **+** | **+** | **+** | **+** | **+** | **+** | **+** | **+** | **+** |
|  |  | ***Sulfolobus solfataricus*** | **+** | **+** | **+** | **+** | **+** | **+** | **+** | **+** | **+** | **+** | **+** |
|  |  | ***Sulfolobus tokodaii str. 7*** | **+** | **+** | **+** | **+** | **+** | **+** | **+** | **+** | **+** | **+** | **+** |
|  | **Thermoproteales （7）** | ***Caldivirga maquilingensis IC-167*** | **+** | **+** | **+** | **+** | **+** | **+** | **+** | **+** | **+** | **+** | **+** |
|  |  | ***Pyrobaculum aerophilum str. IM2*** | **+** | **+** | **+** | **+** | **+** | **+** | **+** | **+** | **+** | **+** | **+** |
|  |  | ***Pyrobaculum arsenaticum DSM 13514*** | **+** | **+** | **+** | **+** | **+** | **+** | **+** | **+** | **+** | **+** | **+** |
|  |  | ***Pyrobaculum calidifontis JCM 11548*** | **+** | **+** | **+** | **+** | **+** | **+** | **+** | **+** | **+** | **+** | **+** |
|  |  | ***Pyrobaculum islandicum DSM 4184*** | **+** | **+** | **+** | **+** | **+** | **+** | **+** | **+** | **+** | **+** | **+** |
|  |  | ***Thermofilum pendens Hrk 5*** | **+** | **+** | **+** | **+** | **-** | **+** | **+** | **+** | **+** | **+** | **+** |
|  |  | ***Thermoproteus neutrophilus V24Sta*** | **+** | **+** | **+** | **+** | **+** | **+** | **+** | **+** | **+** | **+** | **+** |
| **Euryarchaeota** | **Archaeoglobales （3）** | ***Archaeoglobus fulgidus DSM 4304*** | **+** | **+** | **+** | **+** | **+** | **+** | **+** | **+** | **+** | **+** | **+** |
|  |  | [***Archaeoglobus profundus DSM 5631***](http://www.ncbi.nlm.nih.gov/entrez/query.fcgi?db=genomeprj&cmd=PureSearch&details_term=txid572546%5Borgn%5D AND pt_default%5Bprop%5D ) | **+** | **+** | **+** | **+** | **+** | **+** | **+** | **+** | **+** | **+** | **+** |
|  |  | [***Ferroglobus placidus DSM 10642***](http://www.ncbi.nlm.nih.gov/entrez/query.fcgi?db=genomeprj&cmd=PureSearch&details_term=txid589924%5Borgn%5D AND pt_default%5Bprop%5D ) | **+** | **+** | **+** | **+** | **+** | **+** | **+** | **+** | **+** | **+** | **+** |
|  | **Halobacteriales （10）** | ***Haloarcula marismortui ATCC 43049*** | **+** | **+** | **+** | **+** | **-** | **+** | **-** | **+** | **+** | **+** | **+** |
|  |  | ***Halobacterium salinarum*** | **+** | **+** | **+** | **+** | **-** | **-** | **-** | **+** | **+** | **+** | **+** |
|  |  | [***Haloferax volcanii DS2***](http://www.ncbi.nlm.nih.gov/entrez/query.fcgi?db=genomeprj&cmd=PureSearch&details_term=txid309800%5Borgn%5D AND pt_default%5Bprop%5D ) | **+** | **+** | **+** | **+** | **-** | **+** | **-** | **+** | **+** | **+** | **+** |
|  |  | ***Halomicrobium mukohataei DSM 12286*** | **+** | **+** | **+** | **+** | **-** | **+** | **-** | **+** | **+** | **+** | **+** |
|  |  | ***Haloquadratum walsbyi DSM 16790*** | **+** | **+** | **+** | **+** | **-** | **+** | **-** | **+** | **+** | **+** | **+** |
|  |  | ***Halorhabdus utahensis DSM 12940*** | **+** | **+** | **+** | **+** | **-** | **+** | **-** | **+** | **+** | **+** | **+** |
|  |  | ***Halorubrum lacusprofundi ATCC 49239*** | **+** | **+** | **+** | **+** | **-** | **+** | **-** | **+** | **+** | **+** | **+** |
|  |  | [***Haloterrigena turkmenica DSM 5511***](http://www.ncbi.nlm.nih.gov/entrez/query.fcgi?db=genomeprj&cmd=PureSearch&details_term=txid543526%5Borgn%5D AND pt_default%5Bprop%5D ) | **+** | **+** | **+** | **+** | **-** | **+** | **-** | **+** | **+** | **+** | **+** |
|  |  | [***Natrialba magadii ATCC 43099***](http://www.ncbi.nlm.nih.gov/entrez/query.fcgi?db=genomeprj&cmd=PureSearch&details_term=txid547559%5Borgn%5D AND pt_default%5Bprop%5D ) | **+** | **+** | **+** | **+** | **-** | **+** | **-** | **+** | **+** | **+** | **+** |
|  |  | ***Natronomonas pharaonis DSM 2160*** | **+** | **+** | **+** | **+** | **-** | **+** | **-** | **+** | **+** | **+** | **+** |
|  | **Methanobacteriales （4）** | [***Methanobrevibacter ruminantium M1***](http://www.ncbi.nlm.nih.gov/entrez/query.fcgi?db=genomeprj&cmd=PureSearch&details_term=txid634498%5Borgn%5D AND pt_default%5Bprop%5D ) | **+** | **+** | **+** | **+** | **+** | **+** | **-** | **+** | **+** | **+** | **+** |
|  |  | ***Methanobrevibacter smithii*** | **+** | **+** | **+** | **+** | **+** | **+** | **-** | **+** | **+** | **+** | **+** |
|  |  | ***Methanosphaera stadtmanae DSM 3091*** | **+** | **+** | **+** | **+** | **+** | **+** | **-** | **+** | **+** | **+** | **+** |
|  |  | ***Methanothermobacter thermautotrophicus str. Delta*** | **+** | **+** | **+** | **+** | **+** | **+** | **-** | **+** | **+** | **+** | **+** |
|  | **Methanococcales （8）** | ***Methanocaldococcus fervens AG86*** | **+** | **+** | **+** | **+** | **+** | **+** | **+** | **+** | **+** | **+** | **+** |
|  |  | [***Methanocaldococcus infernus ME***](http://www.ncbi.nlm.nih.gov/entrez/query.fcgi?db=genomeprj&cmd=PureSearch&details_term=txid573063%5Borgn%5D AND pt_default%5Bprop%5D ) | **+** | **+** | **+** | **+** | **+** | **+** | **+** | **+** | **+** | **+** | **+** |
|  |  | ***Methanocaldococcus jannaschii DSM 2661*** | **+** | **+** | **+** | **+** | **+** | **+** | **+** | **+** | **+** | **+** | **+** |
|  |  | [***Methanocaldococcus sp. FS406-22***](http://www.ncbi.nlm.nih.gov/entrez/query.fcgi?db=genomeprj&cmd=PureSearch&details_term=txid644281%5Borgn%5D AND pt_default%5Bprop%5D ) | **+** | **+** | **+** | **+** | **-** | **+** | **+** | **+** | **+** | **+** | **+** |
|  |  | ***Methanocaldococcus vulcanius M7*** | **+** | **+** | **+** | **+** | **+** | **+** | **+** | **+** | **+** | **+** | **+** |
|  |  | ***Methanococcus aeolicus Nankai-3*** | **+** | **+** | **+** | **+** | **+** | **+** | **-** | **+** | **+** | **+** | **+** |
|  |  | ***Methanococcus maripaludis*** | **+** | **+** | **+** | **+** | **+** | **+** | **-** | **+** | **+** | **+** | **+** |
|  |  | ***Methanococcus vannielii SB*** | **+** | **+** | **+** | **+** | **+** | **+** | **-** | **+** | **+** | **+** | **+** |
|  | **Methanomicrobiales （5）** | ***Candidatus Methanoregula boonei 6A8*** | **+** | **+** | **+** | **+** | **-** | **+** | **-** | **+** | **+** | **+** | **+** |
|  |  | ***Methanocorpusculum labreanum Z*** | **+** | **+** | **+** | **+** | **-** | **-** | **-** | **+** | **+** | **+** | **+** |
|  |  | ***Methanoculleus marisnigri JR1*** | **+** | **+** | **+** | **+** | **-** | **+** | **-** | **+** | **+** | **+** | **+** |
|  |  | ***Methanosphaerula palustris E1-9c*** | **+** | **+** | **+** | **+** | **-** | **-** | **-** | **+** | **+** | **+** | **+** |
|  |  | ***Methanospirillum hungatei JF-1*** | **+** | **+** | **+** | **+** | **-** | **+** | **-** | **+** | **+** | **+** | **+** |
|  | **Methanopyrales** | ***Methanopyrus kandleri AV19*** | **+** | **+** | **+** | **+** | **+** | **+** | **+** | **+** | **+** | **+** | **+** |
|  | **Methanosarcinales （6）** | ***Methanococcoides burtonii DSM 6242*** | **+** | **+** | **+** | **+** | **+** | **+** | **-** | **+** | **+** | **+** | **+** |
|  |  | [***Methanohalophilus mahii DSM 5219***](http://www.ncbi.nlm.nih.gov/entrez/query.fcgi?db=genomeprj&cmd=PureSearch&details_term=txid547558%5Borgn%5D AND pt_default%5Bprop%5D ) | **+** | **+** | **+** | **+** | **-** | **+** | **-** | **+** | **+** | **+** | **+** |
|  |  | ***Methanosaeta thermophila PT*** | **+** | **+** | **+** | **+** | **-** | **+** | **+** | **+** | **+** | **+** | **+** |
|  |  | ***Methanosarcina acetivorans C2A*** | **+** | **+** | **+** | **+** | **+** | **+** | **-** | **+** | **+** | **+** | **+** |
|  |  | ***Methanosarcina barkeri str. Fusaro*** | **+** | **+** | **+** | **+** | **+** | **+** | **-** | **+** | **+** | **+** | **+** |
|  |  | ***Methanosarcina mazei Go1*** | **+** | **+** | **+** | **+** | **+** | **+** | **-** | **+** | **+** | **+** | **+** |
|  | **Thermococcales （7）** | ***Pyrococcus abyssi GE5*** | **+** | **+** | **+** | **+** | **+** | **+** | **+** | **+** | **+** | **+** | **+** |
|  |  | ***Pyrococcus furiosus DSM 3638*** | **+** | **+** | **+** | **+** | **+** | **+** | **+** | **+** | **+** | **+** | **+** |
|  |  | ***Pyrococcus horikoshii OT3*** | **+** | **+** | **+** | **+** | **+** | **+** | **+** | **+** | **+** | **+** | **+** |
|  |  | ***Thermococcus gammatolerans EJ3*** | **+** | **+** | **+** | **+** | **+** | **+** | **+** | **+** | **+** | **+** | **+** |
|  |  | ***Thermococcus kodakarensis KOD1*** | **+** | **+** | **+** | **+** | **+** | **+** | **+** | **+** | **+** | **+** | **+** |
|  |  | ***Thermococcus onnurineus NA1*** | **+** | **+** | **+** | **+** | **+** | **+** | **+** | **+** | **+** | **+** | **+** |
|  |  | ***Thermococcus sibiricus MM 739*** | **+** | **+** | **+** | **+** | **+** | **+** | **+** | **+** | **+** | **+** | **+** |
|  | **Thermoplasmales （4）** | [***Ferroplasma acidarmanus fer1***](http://www.ncbi.nlm.nih.gov/entrez/query.fcgi?db=genomeprj&cmd=PureSearch&details_term=txid333146%5Borgn%5D AND pt_default%5Bprop%5D ) | **+** | **+** | **+** | **+** | **-** | **+** | **+** | **+** | **+** | **+** | **+** |
|  |  | ***Picrophilus torridus DSM 9790*** | **+** | **+** | **+** | **+** | **-** | **+** | **+** | **+** | **+** | **+** | **+** |
|  |  | ***Thermoplasma acidophilum DSM 1728*** | **+** | **+** | **+** | **+** | **-** | **+** | **+** | **+** | **+** | **+** | **+** |
|  |  | ***Thermoplasma volcanium GSS1*** | **+** | **+** | **+** | **+** | **-** | **+** | **+** | **+** | **+** | **+** | **+** |
| **Nanoarchaeota** | | ***Nanoarchaeum equitans Kin4-M*** | **+** | **+** | **+** | **+** | **-** | **+** | **-** | **+** |  | **+** | **+** |
