# Supplementary material for: Origin and Evolution of the Eukaryotic SSU Processome Revealed by a Comprehensive Genomic Analysis and Implications for the Origin of the Nucleolus
Source: Genome Biol Evol. 2013 Nov 7;5(12):2255–67. doi: 10.1093/gbe/evt173 (PMC3879963; doi:10.1093/gbe/evt173)
Supplement: Supplementary Data [file supp_evt173_suppl_data.zip › Supplementary_data_7.doc]

**Supplementary_data 7. Domain composition of the 26 probable proteins of SSU Processome.**
