# Supplementary material for: Origin and Evolution of the Eukaryotic SSU Processome Revealed by a Comprehensive Genomic Analysis and Implications for the Origin of the Nucleolus
Source: Genome Biol Evol. 2013 Nov 7;5(12):2255–67. doi: 10.1093/gbe/evt173 (PMC3879963; doi:10.1093/gbe/evt173)
Supplement: Supplementary Data [file supp_evt173_suppl_data.zip › Supplementary_data_8.doc]

**8a	8b**

**8c	8d**

**8e**

**Supplementary_data 8. Maximum likelihood phylogenetic tree of the obtained homologous sequences of various SSU processome proteins.** Numbers at branches represent bootstrap proportions. The scale bar represents the average number of substitutions per site. **8a. Phylogenetic tree of the 104 obtained homologous sequences of Nop1;** **8b. Phylogenetic tree of the 164 obtained homologous sequences of Utp23 and Utp24; 8c. Phylogenetic tree of the 89 obtained homologous sequences of Emg1; 8d. Phylogenetic tree of the 119 obtained homologous sequences of Rps4; 8e. Phylogenetic tree of the 117 obtained homologous sequences of Rps6.**
