# Supplementary material for: Origin and Evolution of the Eukaryotic SSU Processome Revealed by a Comprehensive Genomic Analysis and Implications for the Origin of the Nucleolus
Source: Genome Biol Evol. 2013 Nov 7;5(12):2255–67. doi: 10.1093/gbe/evt173 (PMC3879963; doi:10.1093/gbe/evt173)
Supplement: Supplementary Data [file supp_evt173_suppl_data.zip › Supplementary_data_9.doc]

**9a	9b**

**Supplementary_data 9. Maximum likelihood phylogenetic tree of the obtained homologous sequences of three eukaryotic SSU processome proteins.** Numbers at branches represent bootstrap proportions. The scale bar represents the average number of substitutions per site. **9a. Phylogenetic tree of the 222 obtained homologous sequences of Rps9 and Imp3; 9b. Phylogenetic tree of the 121 obtained homologous sequences of Rps14.**
